# Effects of Dietary Corn Type and Coccidiosis Challenge on Broiler Meat Quality

**DOI:** 10.3390/foods15203607

**Published:** 2026-10-10

**Authors:** Jean C. Cáceres, Camila Hammel Sobreira, Jaidyn Abbey, Christina S. Sigmon, Jennifer Rainbow, Samuel Mwangi, Yan L. Campbell

**Affiliations:** Prestage Department of Poultry Science, North Carolina State University, Raleigh, NC 27607, USA

**Keywords:** broiler, corn type, coccidiosis, meat quality

## Abstract

Coccidiosis impairs broiler performance and meat quality, whereas dietary corn source may influence responses to enteric challenges. Therefore, this study evaluated the effects of corn type (yellow corn, YC; orange corn, OC) and coccidiosis challenge (challenged, C+; non-challenged, C-) on broiler (Cobb 500) meat quality. Treatments were arranged in a 2 × 2 factorial RCBD, with eight replicate pens per treatment and 15 birds per pen. The pen was the experimental unit. Breast meat was evaluated for yield, pH, instrumental color, woody breast severity, texture, water-holding capacity, lipid oxidation, and composition. Data were analyzed using PROC GLM in SAS 9.4, with a house section included as a blocking factor. Corn type × challenge interactions were observed for live, chilled, and whole breast weights at 2 h, with YC/C+ showing the lowest values, whereas OC/C+ maintained weights comparable to non-challenged treatments. OC/C+ increased yellowness and had the greatest woody breast incidence, while YC/C+ had the lowest severity. Coccidiosis increased thaw loss and reduced fat content, whereas cook loss, lipid oxidation, and most compositional traits were unaffected. Overall, OC/C+ had greater yield than YC/C+ under challenge but was also associated with greater woody breast severity, indicating an association rather than a causal trade-off.

## 1. Introduction

Coccidiosis is one of the most economically significant parasitic diseases affecting poultry production worldwide. The global economic burden of coccidiosis in chickens has been estimated at approximately US$14.1 billion annually at 2016 prices [1]. It is caused by protozoan parasites of the genus *Eimeria* (phylum *Apicomplexa*), which infect the intestinal epithelium of chickens and result in intestinal tissue damage, impaired nutrient absorption, reduced growth performance, and increased mortality, particularly in broiler chickens between 2 and 8 weeks of age [1]. Clinical manifestations of coccidiosis include diarrhea, decreased feed efficiency, weight loss, reduced carcass yield, and, in severe infections, mortality. Transmission occurs through ingestion of sporulated oocysts present in contaminated litter, feed, water, or equipment.

The pathogenesis of coccidiosis involves invasion of intestinal epithelial cells by *Eimeria* spp., followed by asexual and sexual developmental stages that disrupt intestinal integrity and impair digestive and absorptive functions. The severity and location of intestinal damage vary among *Eimeria* species because each preferentially develops within specific regions of the gastrointestinal tract. In broilers, *E. acervulina* primarily affects the duodenum and proximal small intestine and is associated with enteritis and reduced nutrient absorption, whereas *E. maxima* develops mainly in the jejunum and ileum, where infection can cause epithelial disruption and intestinal wall damage. In contrast, *E. tenella* targets the ceca and can produce severe mucosal damage and hemorrhage [2]. Therefore, the inclusion of *E. acervulina*, *E. maxima*, and *E. tenella* in the challenge provides a model of coccidiosis that affects multiple intestinal regions and represents different degrees and mechanisms of intestinal damage. Collectively, these alterations can compromise nutrient utilization and impair efficiency and growth performance [2,3].

Carotenoids are naturally occurring pigments that play important physiological roles in poultry, including antioxidant activity, immune modulation, and maintenance of tissue pigmentation. Plasma carotenoid concentrations are commonly reduced during coccidial infection due to intestinal damage and decreased absorption efficiency, often resulting in visible depigmentation of the beak, shanks, and skin [4]. Dietary supplementation with carotenoid-rich ingredients has been shown to improve oxidative stability and immune status, maintain pigmentation, and partially alleviate performance losses associated with a coccidiosis challenge [5,6]. Moreover, dietary lutein supplementation has been reported to reduce disease-related responses in broilers challenged with *Eimeria maxima*, suggesting a potential role of carotenoids in mitigating the effects of coccidiosis [7].

Corn is a key energy source in commercial broiler diets and often constitutes a large portion of the formulation due to its high starch content and energy value [8]. However, differences in corn composition and genetic characteristics can influence nutrient and energy utilization by broilers [9]. Orange corn is a promising functional feed ingredient due to its elevated levels of carotenoids, including lutein and zeaxanthin, compared with conventional yellow corn [10]. In the present study, the orange corn diets contained approximately twice the total carotenoid concentration of the yellow corn diets. These carotenoids possess antioxidant properties that may reduce oxidative damage, support epithelial integrity, and enhance immune function during enteric disease challenge. Additionally, carotenoid-rich diets may improve broiler meat quality by preserving color stability and supporting antioxidant capacity in tissues. Supplemental antioxidants, including vitamin E and selenium, have also demonstrated synergistic effects in reducing oxidative stress and supporting intestinal health during periods of pathogenic challenges [11].

In the United States, orange corn has been evaluated as a specialty feed ingredient for poultry, with research examining its potential benefits for animal welfare and product quality, as well as the production scale and costs required for broader industry adoption [12]. Previous work in broilers raised without an experimental coccidiosis challenge showed that orange-corn diets were associated with greater body weight, increased yellow pigmentation of the liver and fat pad, and reduced footpad dermatitis severity compared with conventional corn sources [5]. These findings demonstrate that orange corn can be used as an alternative corn source in broiler diets; however, information regarding its broader application under commercial production conditions remains limited.

Recent evidence suggests dietary composition may influence resistance to coccidiosis and associated intestinal microbial responses [13]. However, limited information is available on the effects of orange corn-based diets on broiler performance and meat quality under coccidiosis-challenge conditions. The present manuscript reports the carcass and meat-quality component of a broader 2 × 2 factorial study evaluating dietary corn source and experimental *Eimeria* challenge in Cobb 500 broilers. The broader study also evaluated production performance, intestinal lesion scores, oocyst enumeration, serum biomarkers, and gene-expression responses, which are being prepared for separate publication [14]. Therefore, the objective of this study was to evaluate the impact of including orange corn in the diet on carcass characteristics and meat quality parameters in broilers subjected to a coccidiosis challenge. It was hypothesized that dietary corn source would influence carcass characteristics and meat quality response of broilers under coccidiosis challenge.

## 2. Materials and Methods

### 2.1. Production

All animal handling, housing, and transport procedures were conducted in accordance with the protocol approved by the Institutional Animal Care and Use Committee (23-256-03). This experiment was conducted between April and May at the NC State University Chicken Education Unit facility. A total of 480 day-old male chicks (Cobb 500) were obtained from a commercial hatchery in North Carolina and transported to the research facility. Chicks were not vaccinated against coccidiosis at the hatchery and did not receive anticoccidial medication before experimental placement. Upon arrival, chicks were randomly distributed to 32 pens, with 15 chicks per pen and 8 replicate pens per treatment. The poultry house was divided into four sections, which were treated as blocks in the statistical analysis. Treatment allocation was randomized within each section such that each of the four dietary corn source × coccidiosis challenge combinations was represented by two replicate pens per section. This blocking structure was used to account for potential variation associated with pen location within the poultry house.

Birds were reared in a curtain-sided poultry house in floor pens measuring approximately 1.8 m *×* 1.2 m, bedded with used wood shavings that were covered with new shavings at placement. Pens had circular pan feeders (36.8 cm diameter; Hog Slat Inc, Newton Grove, NC, USA) and an automatic nipple drinker line. Supplemental feeders and drinkers were provided during the first 7 days postplacement. No additional environmental enrichment was included. Birds were managed according to the recommendations of the Cobb 500 Broiler Management Guide [15]. House temperature was maintained at approximately 32–33 °C at placement and gradually reduced by approximately 2–3 °C per week until reaching 21–22 °C by 21–28 d of age; thereafter, temperature was maintained at approximately 21–24 °C through 42 d. Birds were provided 23 h of light and 1 h of darkness during the first 7 d, followed by 18 h of light and 6 h of darkness until the end of the experiment. Light intensity was maintained at approximately 20–30 lux during brooding (1–14 d) and gradually reduced to 5–10 lux during the grow-out period. During brooding, minimum ventilation was used to control moisture, carbon dioxide, and ammonia while maintaining house temperature. Ventilation rates were progressively increased as birds aged to maintain appropriate environmental conditions and adequate fresh-air exchange. Relative humidity was not recorded; however, environmental conditions were adjusted as needed based on bird behavior and house conditions.

Birds were monitored twice a day throughout the study for general health and signs of distress. Humane endpoints were established in accordance with the approved IACUC protocol. Birds meeting humane endpoint criteria were euthanized by cervical dislocation.

### 2.2. Diets

Two corn-based dietary treatments differing in corn source and natural carotenoid concentration were evaluated: a conventional yellow corn-based diet (YC) and an orange corn-based diet (OC) formulated with naturally carotenoid-enriched orange corn. Each dietary treatment was formulated in two feeding phases: a starter diet from 1 to 21 d of age and a grower diet from 22 to 42 d of age. All starter and grower diets were formulated based on a corn-soybean meal composition. Yellow or orange corn was utilized as the principal cereal ingredient in accordance with the dietary treatment, while soybean meal functioned as the primary oilseed meal. Minor differences in nutrient composition between the yellow and orange corn sources were accounted for during diet formulation to target comparable nutrient specifications between the YC and OC diets within each feeding phase, in accordance with Cobb 500 broiler nutrient recommendations [15]. During the starter phase, YC and OC diets were formulated to contain 22% CP and 2900 kcal/kg AMEn. Analyzed nutrient contents showed CP concentrations of 21.8% and 22.2% and ME values of 3020 and 2976 kcal/kg for the YC and OC diets, respectively. During the grower phase, diets were formulated to contain 18% CP and 3050 kcal/kg AMEn. Analyzed CP concentrations were 17.9% and 18.8%, while analyzed ME values were 3131 and 3064 kcal/kg for the YC and OC diets, respectively.

The full ingredient lists for the experimental starter and grower diets are available in Supplementary Table S1. The analyzed nutrient composition of the finished diets, including crude protein, metabolizable energy, and other components, is shown in Supplementary Table S2. The components analyzed include crude fat, acid detergent fiber, ash, calcium, phosphorus, potassium, magnesium, sodium, iron, manganese, copper, and zinc.

Both starter and grower diets were provided in mash form. The Poultry Vitamin Premix used provided 30,000 IU of vitamin E per pound and was added at 0.05% in starter diets and 0.20% in grower diets. This resulted in approximately 33.1 IU vitamin E/kg in starter feed and 132.3 IU/kg in grower feed. The starter value was below the Cobb 500 supplemental recommendation of 80 IU/kg, whereas the grower value was above the recommendation of 50 IU/kg. No supplemental selenium was included in the diet formulations, as the trace mineral premix lacked selenium. Consequently, supplemental selenium from the premix was 0 mg/kg in the complete feed. However, the overall selenium levels in the complete diets were not measured, and selenium naturally present in the basal ingredients were not quantified. Carotenoid analysis quantified total carotenoids as well as lutein, zeaxanthin, α-cryptoxanthin, β-cryptoxanthin, and β-carotene in each experimental diet. Orange corn diets contained greater total carotenoid concentrations than the corresponding yellow corn diets, with lutein and zeaxanthin representing the predominant measured carotenoids (Table 1). The orange corn starter and grower diets contained 8.12 and 9.96 µg/g total carotenoids, respectively, whereas the yellow corn starter and grower diets contained 3.94 and 4.77 µg/g total carotenoids, respectively. Thus, the orange corn diets provided approximately two-fold greater total carotenoid concentrations than the yellow corn diets throughout the experimental period (Table 1).

### 2.3. Coccidiosis Challenge

At 14 d post-hatch, birds assigned to the challenged groups (C+) were orally inoculated with 0.5 mL of a commercial live coccidiosis vaccine administered at 20× the manufacturer’s recommended dose to induce a controlled *Eimeria* challenge. The vaccine contained live oocysts of *Eimeria acervulina*, *Eimeria maxima*, and *Eimeria tenella*. Birds assigned to the non-challenged groups (C-) were not inoculated. All birds remained on their assigned dietary treatments throughout the experimental period and were processed at 42 d of age. As part of the comprehensive study, intestinal lesion scoring and oocyst enumeration were performed to further characterize the response to the experimental *Eimeria* challenge [14]. On day 21 (7 days post-challenge), the coccidial challenge increased intestinal lesion scores in broilers, regardless of the dietary corn source. Challenged birds fed yellow corn had median lesion scores of 3.0, 2.0, and 2.0 in the jejunum, duodenum, and ceca, respectively, whereas challenged birds fed orange corn had median lesion scores of 3.0, 2.0, and 1.5 in the corresponding intestinal regions. Overall, coccidial challenge significantly increased lesion scores in the jejunum, duodenum, and ceca (*p* < 0.05). However, dietary corn source did not significantly affect lesion scores (*p* > 0.05), and no challenge × diet interaction was detected (*p* > 0.05), indicating that lesion severity was primarily driven by the coccidial challenge rather than by corn source [14].

### 2.4. Processing

The birds were raised until they reached 42 d, to reflect standard commercial practices [15]. Prior to slaughter, birds were subjected to an 8–12 h feed withdrawal period. All animal handling, pre-slaughter, and processing procedures were conducted in accordance with the protocol approved by the Institutional Animal Care and Use Committee (22-402-06). For sampling, one bird was randomly selected from each pen for processing, resulting in 8 sampled birds per treatment. Each bird was tagged with a unique ID number the day before processing and transported to the NC State University Processing Pilot Plant for slaughter and subsequent data collection.

Live broilers were weighed, then stunned with a 15-volt electric knife for 10 s. A deep “V” cut was made to sever the arteries on both sides of the neck, keeping the esophagus and trachea intact, and bleeding for about 2 min. Afterward, the birds were processed in the scalder for approximately 60 s at 62.8 ± 2 °C, followed by feather removal in the plucker. Next, the broilers were taken off the line, and their internal organs were manually extracted using a knife on stainless steel tables. All weight measurements were obtained with a digital scale (Ohaus, Champ Scale BASE With CD-11 Weighing Indicator Terminal, Parsippany, NJ, USA). The carcasses were kept in a chiller tank for 90 min at temperatures ranging from 0 to 4 °C, with the temperature monitored periodically using a thermometer (THERMO-WORKS, RT301WA-N, American Fork, UT, USA) to ensure it remained within range.

After chilling, the carcasses were transferred to the cutting room at 10 °C, and their post-chill weights were recorded. Each carcass was sectioned into primal cuts, such as breasts, tenders, wings, thighs, and drumsticks. Weights were recorded only for the whole and the right breast. The pH was measured 2 h postmortem in the right breast using a portable pH meter (HANNA Instrument, HI98163, Smithfield, RI, USA). After processing, cut-up parts were placed in labeled Ziploc bags and stored at 2–4 °C until 24 h postmortem.

### 2.5. Myopathic Conditions Scoring

The woody breast (WB) scores were recorded after weights were collected 2 h postmortem. For WB assessment, a trained lab member palpated the breasts to evaluate hardness, following standard procedures used in poultry meat quality studies [16,17]. The severity was rated on a 4-point scale: 0 for “normal,” 1 for “mild” (hardness in the cranial region), 2 for “moderate” (hardness throughout the breast but with some flexibility), and 3 for “severe” (more than half of the breast was firm).

### 2.6. 24 h Post-Processing

After 24 h postmortem, the breasts were removed from the Ziploc bags. pH and color were measured at this point using a portable pH meter (HANNA Instrument, HI98163, Smithfield, RI, USA) and a portable color meter (KONICA MINOLTA, CR-400, Ramsey, NJ, USA) at three different points on the breast, sectioned as top, middle, and bottom. Once data collection was complete, the pieces of interest were placed in pre-labeled, vacuum-sealed bags (BUNZL, Clarity 3-mil, Winston-Salem, NC, USA) for subsequent analysis. A vacuum sealer (ULTRASOURCE, 500, Kansas City, MO, USA) was used to seal the bags, and the sealed bags were stored at −20 °C. Samples remained continuously frozen at −20 °C until analysis, with no known thawing events or substantial temperature fluctuations during storage.

### 2.7. Meat Quality After 5 Months of Frozen Storage

The following analyses were performed using right breast samples collected from 8 replicate pens per treatment. The 5 mo storage period was selected to represent an extended frozen storage interval prior to meat-quality evaluation. After 5 mo of storage, meat samples from the right and left breasts were distributed for various analyses, including pH, color, meat composition using near-infrared (NIR) spectroscopy, and lipid oxidation using thiobarbituric acid reactive substances (TBARS). Thaw and cook loss and texture were performed on the left breast. These analyses were performed consecutively on two days in November 2025.

### 2.8. pH

pH was measured using a portable pH meter equipped with a penetration probe (HANNA Instrument, model HI98163, Smithfield, RI, USA), calibrated with 4.01 and 7.01 buffer solutions. The measurements were consistently taken from the upper right side of the breast.

### 2.9. Color

Color was collected at 24 h and 5 months. The Chroma Meter CR-400 (KONICA MINOLTA, CR-400, Ramsey, NJ, USA; D65 illuminant, 2° standard observer angle) was calibrated with a white plate, yielding Y = 93.5, x = 0.311, and y = 0.319. The instrument was paired with SpectraMagicTM NX (KONICA MINOLTA, CM-S100w, version 3.40, Ramsey, NJ, USA). Readings were expressed using the Commission Internationale de l’Éclairage (CIE) L*a*b* color system [18]. Readings were taken at three points on the breast: top (cranial), middle (medial), and bottom (caudal), as previously described.

### 2.10. Meat Composition (NIR)

Samples for pH and color analyses were processed and ground with a food processor (Black &Decker, model FP4200, Towson, MD, USA). Two samples were extracted: one for composition analysis, and the remaining was stored at −80 °C. Near-infrared (NIR) analysis was conducted using a FoodScan TM 2 Meat Analyzer with ISIScanTM Nova Software (FoodScanTM 2, FOSS Analytical A/S, version 10.2.1.13, Hillerød, Denmark) to assess meat composition, including fat, protein, collagen, moisture, and ash, following AOAC Official Method 2007.04 [19].

### 2.11. Lipid Oxidation

The TBARS assay, adapted from Buege and Aust [20] and described by Luqué et al. [21], was used to measure lipid oxidation in meat by assessing malondialdehyde (MDA) levels. About 20 g of representative samples stored at −80 °C were ground into a fine powder using a coffee blender (SECURA, Sandy, UT, USA) with Nitrogen (Airgas, Radnor, PA, USA). A 5 g portion was placed in a 50 mL conical tube. For lipid extraction, 15 mL of cold deionized water was added, followed by homogenization at 10,000 rpm with a 20 mm probe for 30 s and vortexing for 30 s. The samples were centrifuged at 1850× *g* for 10 min; 2 mL of supernatant was transferred to a new tube, to which 4 mL of TBA/TCA reagent (15%TCA *w*/*v* and 20 mM TBA; Sigma-Aldrich, St. Louis, MO, USA) and 100 µL of BHA solution (Sigma-Aldrich, St. Louis, MO, USA) dissolved in 90%ethanol were added. After vortexing for 1 min, samples were heated at 100 °C for 15 min in a water bath (Fisher Scientific, Isotemp GDP 20, Pittsburgh, PA, USA), then cooled on ice for 10 min. Following cooling, a second centrifugation was performed under the same conditions, and 200 µL of supernatant was transferred in duplicate to a 96-well plate. A standard curve was prepared using serial dilutions of tetraethoxypropane (TEP; Sigma-Aldrich, St. Louis, MO, USA). Absorbance was measured at 531 nm with a plate reader (Molecular Devices, SpectraMax^®^ i3x, San Jose, CA, USA). The standard curve’s intercept and slope, determined through linear regression, were used to calculate the MDA concentration in the samples.

A review of the results was conducted, and samples with a coefficient of variation (CV) greater than 5% between duplicates were reanalyzed to improve accuracy.

### 2.12. Thaw and Cook Loss

One left breast sample was collected from each carcass in each of the 8 replicates per treatment. These samples were evenly spread on pan sheets and refrigerated for 24 h to thaw. They were weighed initially while frozen and then again after thawing. The weight loss was calculated based on these measurements:

Thaw loss (%) = ((Frozen Weight − Thawed Weight)/Frozen Weight) × 100(1)

To determine cooking loss, all 8 left-side breasts were evenly distributed on oven racks and cooked in a convection oven (WOLF, model IR36550/S/T, Fitchburg, WI, USA) at 176.7 °C until the internal temperature reached 74 °C. The following formula was used to calculate cooking loss:

Cook loss (%) = ((Raw Weight − After Cooking Weight)/Raw Weight) × 100(2)

### 2.13. Texture BMORS and Warner-Bratzler

For texture analysis, a texture analyzer (Stable Micro Systems, TA.XT plus C, Godalming, UK) was used with a 30 kg load cell. The Meullenet-Owens razor shear (MORS) method was employed, specifically the blunt-blade version (BMORS). One breast from each of the 8 sampled carcasses per treatment was used for texture analysis. The test parameters were set to 2 mm/s speed and 5 g trigger force. Tests were conducted on raw breasts, with measurements taken in triplicate in the upper right corner of each. Afterward, the samples were evenly placed on baking pans and cooked in a convection oven (WOLF, model IR36550/S/T, Fitchburg, WI, USA) at 176.7 °C until the internal temperature reached 74 °C. The breasts were then removed and cooled for five minutes. The same test procedure was repeated using BMORS. For the Warner-Bratzler test, after the initial test, the samples were cut into 1.5 cm × 1.5 cm × 2 cm pieces, and the peak shear force (N) and total energy (N · mm) were measured. Each sample was tested in triplicate, and the average values were used for statistical analysis.

### 2.14. Statistical Analysis

A 2 *×* 2 factorial arrangement of treatments was used to evaluate the effects of dietary corn source (yellow corn vs. orange corn) and coccidiosis challenge status (challenged vs. non-challenged) on carcass characteristics and meat quality traits. The four treatment combinations were yellow corn/non-challenged (YC/C-), orange corn/non-challenged (OC/C-), yellow corn/challenged (YC/C+), and orange corn/challenged (OC/C+). All statistical analyses were conducted using SAS software (SAS Institute Inc., version 9.4, Cary, NC, USA). Pen served as the experimental unit, with eight replicate pens per treatment. For carcass and meat-quality evaluations, one bird was randomly selected from each pen; thus, the sampled bird represented the observational unit within its respective experimental pen, yielding eight independent pen-level observations per treatment.

No a priori inclusion or exclusion criteria were established beyond the experimental design and sampling procedures. No animals, experimental units, or data points were excluded from the analyses. Unless otherwise stated, analyses included eight replicate pens per treatment. No single primary outcome measure was designated for sample size determination because the study was designed to evaluate multiple carcass and meat quality responses within the same factorial experimental design.

Continuous carcass and meat-quality responses were analyzed using PROC GLM in SAS 9.4. House section was included as a blocking factor, while dietary corn source, coccidiosis challenge, and their interaction (Corn type × Challenge) were included as treatment effects. Least-squares means were generated for corn type, coccidiosis challenge, and the Corn type × Challenge interaction. When a significant interaction was detected (*p* ≤ 0.05), pairwise comparisons among treatment-combination least-squares means were performed using the PDIFF option. When the interaction was not significant, the main effects of corn type and coccidiosis challenge were interpreted from the corresponding least-squares means and Type III tests of fixed effects from the GLM. Statistical significance was declared at *p* ≤ 0.05. Measurements collected at 24 h and after 5 mo of frozen storage were analyzed separately; storage time was not included as a repeated factor, and no statistical comparisons between the two time points were performed.

Woody breast severity was assessed using an ordered numerical scale from 0 to 3 and was analyzed using the same block-adjusted 2 × 2 factorial GLM to evaluate the effects of corn type, coccidiosis challenge, and their interaction. Because WB scores represent ordered severity categories, a Kruskal–Wallis test was additionally performed across the four treatment combinations as a nonparametric sensitivity analysis. The Kruskal–Wallis analysis was used to evaluate whether the overall distribution of WB scores differed among treatment combinations, whereas the factorial GLM was retained to evaluate the corn type × coccidiosis challenge interaction.

No blinding was implemented during animal allocation, conduct of the experiment, outcome assessment, or statistical analysis. Treatment assignments were known to the personnel involved throughout the study.

## 3. Results

### 3.1. Carcass and Breast Meat Yield

Corn type × coccidiosis challenge interactions were observed for live, chilled, whole breast weight at 2 h (*p* < 0.05; Table 2). In this section, treatments are referred to as yellow corn (YC), orange corn (OC), coccidiosis-challenged (C+), and non-challenged (C-). For live weight, YC/C+ had the lowest value at 3.20 kg, whereas OC/C-, OC/C+, and YC/C- had greater values of 3.40, 3.44, and 3.49 kg, respectively (*p* = 0.024). A similar pattern was observed for chilled weight, where YC/C+ had the lowest value at 2.43 kg, compared with 2.71 kg for OC/C-, 2.76 kg for OC/C+, and 2.79 kg for YC/C- (*p* = 0.003).

For whole breast weight at 2 h, YC/C+ had the lowest value at 0.67 kg, while OC/C-, OC/C+, and YC/C- had values of 0.77, 0.79, and 0.79 kg, respectively (*p* = 0.043). At 24 h, the corn type × coccidiosis challenge interaction for whole breast weight was not significant (*p* = 0.053).

No corn type *×* coccidiosis challenge interactions were observed for right breast weight at 2 h, right breast weight at 24 h, or right breast weight after 5 mo of frozen storage (*p* = 0.064, *p* = 0.086, and *p* = 0.851, respectively). Additionally, for the same traits, none of the main effects had an influence (*p* > 0.05). Weights remained within the ranges of 0.34 to 0.40 kg, 0.33 to 0.39 kg, and 0.30 to 0.33 kg, respectively.

### 3.2. Postmortem and Post-Storage pH

No corn type × coccidiosis challenge interactions were observed for pH at any time point (2 h and 24 h postmortem, and 5 mo of frozen storage; *p* > 0.05; Table 3). At 2 h, no effect of corn type or coccidiosis challenge was observed (*p* = 0.131 and *p* = 0.292, respectively). Similarly, at 24 h, no effect of corn type or coccidiosis challenge was detected (*p* = 0.991 and *p* = 0.934, respectively).

After 5 mo of frozen storage, the coccidiosis challenge did not affect pH (*p* = 0.440), whereas corn type had an influence, with breast meat from YC exhibiting a greater pH than OC breast meat (6.23 vs. 6.12, respectively; *p* = 0.019).

### 3.3. Postmortem and Post-Storage Instrumental Color (L*a*b*)

At 24 h postmortem, no corn type × coccidiosis challenge interaction was observed for lightness (L*) and redness (a*) (*p* = 0.117 and *p* = 0.111, respectively; Table 4). However, a corn type *×* coccidiosis challenge interaction was observed for yellowness (b*; *p* = 0.044). Breast meat from OC/C+ was yellower than YC/C+ (12.67 vs. 10.12), whereas OC/C- and YC/C- had intermediate b* values. No main effects of corn type or coccidiosis challenge were observed for L* (*p* = 0.795 and *p* = 0.115, respectively) or a* (*p* = 0.115 and *p* = 0.928, respectively) at 24 h postmortem.

After 5 months of frozen storage, corn type × coccidiosis challenge interactions were observed for lightness (L*) and yellowness (b*) (*p* = 0.013 and *p* = 0.040, respectively). For lightness, YC/C- samples were brighter than YC/C+ samples (60.98 vs. 57.54), whereas OC/C- and OC/C+ samples were intermediate. For yellowness after storage, OC/C+ samples were yellower (11.41) than YC/C- (9.65) and YC/C+ (8.91), whereas OC/C- (10.06) was intermediate. Redness (a*) after 5 months was affected by corn type, with OC samples exhibiting greater redness than YC samples (0.95 vs. −0.01, respectively; *p* = 0.029). However, the coccidiosis challenge did not affect redness after storage (*p* = 0.686).

### 3.4. Woody Breast Incidence and Severity

A corn type × coccidiosis challenge interaction was observed for WB severity score (*p* = 0.013; Table 5). WB severity scores were greater in OC/C+ and YC/C- than in YC/C+, whereas OC/C- was intermediate. Mean WB severity scores were 2.38, 2.25, 1.88, and 1.00 for OC/C+, YC/C-, OC/C-, and YC/C+, respectively. The nonparametric sensitivity analysis also detected an overall difference in WB severity across the four treatment combinations (Kruskal–Wallis χ^2^ = 9.93, df = 3, *p* = 0.0192), supporting the overall treatment pattern observed in the factorial analysis.

The distribution of WB scores also varied among treatments (Table 6). In the absence of a coccidiosis challenge, all breasts from the YC/C- treatment exhibited some degree of WB (8/8; 100%), with most samples classified as moderate (WB2; 50.0%) or severe (WB3; 37.5%). Similarly, 87.5% (7/8) of breasts from the OC/C- treatment were affected, with moderate WB (WB2) representing the most common classification (50.0%).

Under coccidiosis challenge conditions, WB incidence remained high in both corn types, although severity differed. In the YC/C+ treatment, 75.0% (6/8) of breasts were affected, and all cases were classified as mild (WB1; 50.0%) or moderate (WB2; 25.0%), with no severe cases observed. In contrast, 87.5% (7/8) of breasts from the OC/C+ treatment exhibited WB, including the greatest proportion of severe cases among all treatments (WB3; 62.5%). The remaining samples affected were classified as moderate (WB2; 25.0%).

### 3.5. Instrumental Texture Properties

Warner-Bratzler force (N) was not affected by corn type × coccidiosis challenge interaction (*p* = 0.177), corn type (*p* = 0.799), or coccidiosis challenge (*p* = 0.378; Table 7). However, a corn type × coccidiosis challenge interaction was observed for toughness (*p* = 0.019). OC/C- had greater toughness than OC/C+ and YC/C-, whereas YC/C+ was intermediate.

In contrast, BMORS traits were affected by corn type × coccidiosis challenge interactions. For raw shear energy, YC/C- was greater than YC/C+ (172.10 vs. 95.29 N·mm), whereas OC/C- and OC/C+ were intermediate (*p* = 0.039). Cooked shear energy was not affected by corn type, coccidiosis challenge, or their interaction (*p* > 0.05).

For raw firmness, YC/C- had a greater value than YC/C+ (19.75 vs. 10.73 N), whereas OC/C- and OC/C+ were intermediate (*p* = 0.043). Cooked firmness was not affected by corn type, coccidiosis challenge, or their interaction (*p* > 0.05). Raw peak count was not affected by corn type, coccidiosis challenge, or their interaction (*p* > 0.05). Cooked peak count was not affected by corn type, coccidiosis challenge, or their interaction (*p* > 0.05; Table 7).

### 3.6. Water-Holding Capacity

No corn type × coccidiosis challenge interaction was observed for thaw loss or cook loss (*p* = 0.934 and *p* = 0.504, respectively; Table 8). Cook loss was not affected by corn type or coccidiosis challenge (*p* = 0.592 and *p* = 0.837, respectively). Corn type effect values for cook loss ranged from 29.21% to 30.49%, whereas coccidiosis challenge values ranged from 29.60% to 30.10%.

In contrast, thaw loss was affected by coccidiosis challenge (*p* = 0.031). Breast from C+ had a greater thaw loss percentage than breast meat from C- (7.40 vs. 6.29%). Corn type did not affect thaw loss (*p* = 0.178), and values ranged from 6.51 to 7.18%.

### 3.7. Lipid Oxidation

Lipid oxidation, expressed as mg MDA/kg of meat, was not affected by corn type × coccidiosis challenge interaction (*p* = 0.079; Table 8), corn type (*p* = 0.552), or coccidiosis challenge (*p* = 0.319). Treatment values were 0.54 mg MDA/kg for OC/C-, 0.61 mg MDA/kg for OC/C+, 0.64 mg MDA/kg for YC/C-, and 0.39 mg MDA/kg for YC/C+.

### 3.8. Proximate Meat Composition

A corn type × coccidiosis challenge interaction was observed for protein content (*p* = 0.043), whereas no interactions were observed for fat, moisture, collagen, or ash content (*p* > 0.05; Table 9). No corn-type effects were observed for fat, moisture, protein, collagen, or ash content (*p* > 0.05). Coccidiosis challenge did not affect moisture, protein, collagen, or ash content (*p* > 0.05). However, fat content was influenced by coccidiosis challenge (*p* = 0.018). Breast meat from C+ had a lower fat content than that from C- (2.20 vs. 2.77%). Despite the significant interaction for protein, pairwise comparisons among treatment combinations were not significant (*p* > 0.05).

## 4. Discussion

### 4.1. Carcass and Breast Meat Yield

The interaction observed for live weight, chilled weight, and whole breast weight at 2 h indicates that the effect of coccidiosis challenge on live weight and breast meat yield depended on the dietary corn source. As reported in the Results section, breast meat from YC/C+ had the lowest live weight, chilled weight, and whole-breast weight at 2 h, whereas breast meat from OC/C+ had values comparable to those of both non-challenged treatments (Table 2).

The responses of live weight and carcass-related traits to the experimental challenge varied by dietary corn source, with lower values observed in YC/C+ compared to other treatments. Similar findings have been reported in previous studies, showing reduced growth performance and feed efficiency following coccidial infection [22,23].

The greater live and breast meat weights observed in OC/C+ compared with YC/C+ indicate that the response to the experimental challenge differed according to dietary corn source. The OC diets contained approximately twice the total carotenoid concentration of the YC diets and differed in their individual carotenoid profiles (Table 1). Previous research has associated orange-corn diets with differences in growth and tissue pigmentation in broilers [5]. However, because the experimental factor was the corn source rather than isolated carotenoid supplementation, the observed responses cannot be attributed specifically to carotenoids. Other unmeasured compositional or phytochemical differences between the corn sources may also have contributed. Furthermore, carotenoid concentrations in plasma or tissues and antioxidant status were not measured; therefore, the biological mechanism underlying the corn-source response cannot be determined from this study.

The reduction in breast meat yield observed in YC/C+ may also be biologically connected to the lower WB severity observed in that treatment. Severe WB is often associated with heavier birds, greater breast weight, and increased breast muscle thickness [24,25]. Thus, the lower yield of YC/C+ may have been associated with reduced growth pressure on the pectoralis major muscle and the lower WB severity observed in this treatment. In contrast, OC/C+ maintained greater breast yield under challenge, but this response coincided with the highest WB severity score and the greatest proportion of WB3 cases (Table 2, Table 5 and Table 6). This indicates an observed association between greater breast meat yield and greater WB severity under the conditions of this study.

### 4.2. Postmortem and Post-Storage pH

The lack of treatment effects on pH at 2 h and 24 h postmortem suggests that neither corn type nor coccidiosis challenge markedly altered early postmortem glycolysis in the breast muscle (Table 3). These results align with the typical decrease in pH observed during the onset of rigor mortis [26]. Postmortem pH decline is primarily driven by glycogen metabolism and lactic acid accumulation, and differences in ultimate pH can influence color, water-holding capacity, and processing functionality [27,28]. Therefore, the absence of pH differences at 2 h and 24 h suggests that the treatment effects observed in yield, color, WB severity, and BMORS texture were not primarily attributable to early postmortem pH differences.

After 5 mo of frozen storage, YC breast meat had a greater pH than OC breast meat, although the difference was relatively small (6.23 vs. 6.12; Table 3), and both remained within normal pH ranges for poultry meat [29,30]. Frozen storage can affect poultry meat quality through protein denaturation, water redistribution, and changes in color and texture, but pH responses during frozen storage are often less consistent than those for water loss or tenderness [31]. In the present study, the higher post-storage pH in YC did not correspond to lower thaw loss because thaw loss was affected by coccidiosis challenge rather than corn type. This suggests that post-storage pH alone was not the main driver of water-holding capacity responses in this study.

### 4.3. Postmortem and Post-Storage Instrumental Color (L*a*b*)

The effect of dietary corn source on meat yellowness was most evident under the coccidiosis challenge. At 24 h postmortem, OC/C+ breast meat exhibited greater b* values than YC/C+, whereas OC/C- and YC/C- were intermediate. After 5 mo of frozen storage, OC/C+ remained among the treatments with the greatest yellowness (Table 4). Orange corn contains greater concentrations of carotenoids and xanthophylls than conventional yellow corn [32], and previous studies have reported increased pigmentation in tissues such as liver, fat pad, skin, and egg yolk following consumption of carotenoid-rich corn [5,32]. Although tissue samples were collected during the present experiment, carotenoid analyses have not yet been performed. Therefore, the greater b* values observed in OC treatments should be interpreted as an association with dietary corn source rather than as direct evidence of increased carotenoid deposition in breast muscle.

After 5 mo of frozen storage, the L* and b* interactions indicate that storage may have amplified or revealed treatment-dependent differences in color. Previous studies have reported that frozen broiler breast fillets became darker, redder, and less yellow as the duration of frozen storage increased, indicating that freezing can modify color attributes over time [31]. In the current study, OC/C+ exhibited greater yellowness after storage than YC/C- and YC/C+, whereas YC/C- had greater L* values than YC/C+ (Table 4). These results suggest that the combination of dietary corn and disease challenge influences post-storage color traits. Since orange corn diets had higher total carotenoid levels compared to yellow corn diets, the greater yellowness observed in OC/C+ was associated with the higher carotenoid content of the OC diet. However, because carotenoid levels in breast meat and the meat’s oxidative status were not measured, the exact mechanism remains uncertain.

The greater a* values observed in OC compared to YC after 5 months of storage suggest a corn-source-related difference in redness; however, this mechanism cannot be determined from the current study. Given the generally low levels of redness, the biological significance of this difference should be interpreted with caution. Additionally, the most consistent color response in this study was a greater yellowness of OC/C+ breast meat compared with the other treatments, particularly after storage.

### 4.4. Woody Breast Incidence and Severity

The results show that corn × coccidiosis challenge interaction influenced myopathy severity. OC/C+ and YC/C- had greater average WB severity scores than YC/C+, while OC/C- was intermediate (Table 5). OC/C+ also had the most severe WB cases, with 62.5% of breasts rated as WB3, while YC/C+ had no severe cases (Table 5). These results suggest that the lower yield in YC/C+ may be related to less severe WB, whereas greater yield in OC/C+ may be associated with more severe WB.

These findings align with earlier studies showing that WB is more common in heavier birds with greater breast weight and thicker breast muscles [24,25,33]. In this study, the OC/C+ group had higher live and breast meat weights than the YC/C+ group during the challenge, and these greater weights coincided with greater WB severity. The higher WB severity observed in YC/C- also supports an association between greater growth or breast yield and WB, as this group had greater live and whole-breast weights at 2 h than YC/C+ (Table 2, Table 5 and Table 6).

The lower WB severity seen in YC/C+ may be due to the challenge, which leads to reduced growth and breast muscle development. Rapid breast muscle growth is associated with poor blood supply, low oxygen levels, oxidative stress, and muscle fiber damage, all key components of WB [25,34]. Because the YC/C+ group had lower live and breast weights, these reductions may have been associated with the lower WB severity observed in this treatment. However, this should not be viewed as a benefit of coccidiosis, but rather as a side effect of reduced growth during the challenge.

In contrast, OC/C+ birds had a greater yield than YC/C+ under the challenge but had the most severe WB cases. This suggests that the greater yield observed in OC/C+ was not associated with lower WB severity. However, given the eight birds assessed per treatment, this relationship should be considered hypothesis-generating rather than evidence of a causal trade-off.

### 4.5. Instrumental Texture Properties

The lack of treatment effects on Warner-Bratzler force indicates that this measurement did not detect differences among treatments, whereas Warner-Bratzler toughness showed a corn type × coccidiosis challenge interaction (Table 7), which is particularly relevant because cooked texture is more representative of the characteristics experienced by the consumer. BMORS measurements also showed corn type × coccidiosis challenge interactions for raw shear energy and firmness. This suggests that BMORS may have captured structural changes in the breast fillets that may not always be noticeable to consumers.

Greater raw BMORS shear energy and firmness were observed in YC/C- than in YC/C+, which corresponded to the greater WB severity observed in YC/C- (Table 6 and Table 7). This association is consistent with WB as a condition involving hardened breast muscle, increased fibrous tissue, and altered physical structure [25,33]. Another study reported that WB meat differed from normal meat in pH, color, cooking loss, composition, and proteome profile, although shear force responses may not always differ across methods [34].

The lower raw BMORS shear energy and firmness observed in YC/C+ compared with YC/C- align with its lower WB severity distribution. Because YC/C+ had no severe WB cases and a lower mean WB score, the reduced BMORS values may reflect a lower degree of muscle hardness and structural alteration.

### 4.6. Water-Holding Capacity

Thaw loss was affected by coccidiosis challenge, with C+ breast meat exhibiting greater thaw loss than C- breast meat (Table 8). This response agrees with previous findings that coccidiosis can impair carcass and meat quality and reduce water-holding capacity, resulting in greater drip or thawing losses [35]. The greater thaw loss in C+ samples may reflect challenge-related changes in muscle structure, protein functionality, or post-storage water retention. Coccidiosis can also impair intestinal integrity and nutrient utilization, potentially indirectly affecting muscle development and meat quality [22].

The absence of a cook loss response suggests that the effect of coccidiosis challenge was more evident during freezing and thawing than during cooking. Frozen storage can disrupt muscle structure through ice crystal formation and water redistribution, resulting in increased thaw loss and reduced moisture retention [31]. Therefore, the greater thaw loss in C+ meat may indicate that tissue from challenged birds was less able to retain water after frozen storage. However, because cook loss did not differ among treatments, the challenge-related difference observed in thaw loss was not also observed during cooking.

### 4.7. Lipid Oxidation

Lipid oxidation was not affected by corn type, coccidiosis challenge, or their interaction (Table 8). These results were unexpected, as coccidiosis has been associated with oxidative stress responses in broilers, including changes in antioxidant enzyme activity and malondialdehyde concentrations [23,35]. However, the lack of TBARS differences suggests that the treatments did not produce measurable differences in lipid oxidation in breast meat under the conditions of this study.

The absence of a corn-type effect also suggests that OC did not significantly improve lipid oxidative stability compared with YC. This may indicate that the carotenoid contribution from OC was insufficient to alter the lipid oxidation rate in breast muscle, or that carotenoids were deposited more strongly in other tissues, such as the fat pad or liver, as previously reported in other studies [5]. However, because carotenoid deposition and antioxidant status were not measured, the contribution of dietary carotenoids to this response cannot be determined from the present study. Additionally, breast meat is relatively lean, which may limit the magnitude of detectable differences in lipid oxidation compared with fattier tissues.

Although the corn type × coccidiosis interaction for TBARS approached significance, it did not meet the statistical threshold. Therefore, the numerical reduction observed in YC/C+ should not be interpreted as a true treatment effect. Instead, these findings indicate that the most evident treatment responses were related to yield, color, WB severity, texture, thaw loss, and fat content rather than lipid oxidation.

### 4.8. Proximate Meat Composition

Coccidiosis challenge reduced fat content but did not affect moisture, collagen, or ash content, whereas protein content showed a corn type × coccidiosis challenge interaction (Table 9). However, pairwise comparisons among treatment combinations did not differ for protein content. This reduction in fat may be related to the metabolic cost of the immune response and impaired nutrient utilization during coccidiosis challenge. Coccidiosis damages the intestinal tract and can reduce nutrient absorption, feed efficiency, and growth performance, potentially shifting nutrient partitioning away from fat deposition [22,23].

The lack of corn-type main effects on proximate composition suggests that replacing YC with OC did not substantially alter the basic chemical composition of breast meat. This is important because OC was associated with selected yield and color traits, without causing major differences in moisture, collagen, or ash. Therefore, the effects of OC in the present study were more evident in selected yield and color traits than in the proximate composition of breast meat.

The reduction in fat content in challenged birds is consistent with the lower carcass yield observed in YC/C+ and may reflect challenge-related changes in nutrient utilization and tissue deposition. However, because the fat reduction was observed as a main effect of coccidiosis rather than a corn type × coccidiosis interaction, the reduction in fat content appears to reflect a generalized response to disease challenge rather than dietary corn type.

### 4.9. Limitations

Meat quality analyses were based on one representative bird from each experimental pen, which may not fully characterize within-pen biological variability. Additionally, corn source, rather than isolated carotenoid supplementation, was the experimental dietary factor, and carotenoid concentrations in plasma, breast muscle, or other tissues, as well as antioxidant status and systemic oxidative markers, were not evaluated in the present meat-quality analysis. Therefore, although the OC diets contained greater carotenoid concentrations, the observed dietary responses cannot be attributed specifically to carotenoid absorption, tissue deposition, or antioxidant activity, and contributions from other unmeasured compositional differences between corn sources cannot be excluded. The present manuscript presents the carcass and meat-quality component of a broader study in which production performance, intestinal lesion scores, oocyst enumeration, serum biomarkers, and gene expression outcomes were also evaluated [14]. Because these additional outcomes are being reported separately, the carcass and meat-quality responses presented here should be interpreted within the specific scope of the present analysis rather than as a complete characterization of the physiological response to the experimental *Eimeria* challenge. The study was conducted using male Cobb 500 under a specific coccidial challenge, dietary program, housing environment, and processing protocol; therefore, the findings should be extrapolated with caution to other strains, sexes, ages, production systems, or challenge conditions.

## 5. Conclusions

Dietary corn source influenced selected carcass and breast meat quality responses in broilers subjected to the experimental coccidiosis challenge. The OC/C+ treatment had greater live, chilled, and whole-breast weights at 2 h than YC/C+, while orange corn was also associated with greater breast meat yellowness under selected challenge and storage conditions. Coccidiosis challenge increased thaw loss and reduced breast meat fat content but had limited effects on several other meat quality traits. Greater woody breast severity in OC/C+ was concurrent with greater breast weight; however, this relationship reflects an observed association rather than a demonstrated causal trade-off. Although the orange corn diets contained approximately twice the total carotenoid concentration of the yellow corn diets, further research is needed to determine the mechanisms underlying these dietary responses and their relevance under commercial broiler production conditions.

## Figures and Tables

**Table 1:** Total and individual carotenoid content (µg/g) of experimental yellow corn (YC) and orange corn (OC) starter and grower diets.

Diet	Feeding Phase	Total Carotenoids (µg/g)	Lutein	Zeaxanthin	α-Cryptoxanthin	β-Cryptoxanthin	β-Carotene
YC	Starter	3.94	1.95	1.16	0.23	0.28	0.32
OC	Starter	8.12	3.54	3.25	0.53	0.46	0.35
YC	Grower	4.77	2.69	1.30	0.26	0.27	0.26
OC	Grower	9.96	4.38	3.89	0.64	0.51	0.54

YC = Yellow corn, OC = Orange corn.

**Table 2:** Weight differences in kg at 2 h and 24 h from different corn diet treatments ^1^.

Factors		Live Weight	Chilled Weight	Whole Breast 2 h	Whole Breast 24 h	Right Breast 2 h	Right Breast 24 h	Right Breast 5 mo
Corn Type	Coccidiosis Challenge							
		Interaction effects						
OC	C-	3.40 ^a^	2.71 ^a^	0.77 ^a^	0.74	0.38	0.37	0.30
	C+	3.44 ^a^	2.76 ^a^	0.79 ^a^	0.75	0.40	0.38	0.30
YC	C-	3.49 ^a^	2.79 ^a^	0.79 ^a^	0.77	0.40	0.39	0.33
	C+	3.20 ^b^	2.43 ^b^	0.67 ^b^	0.65	0.34	0.33	0.32
*SEM* ^2^		0.069	0.061	0.033	0.030	0.018	0.016	0.017
		Main effects						
OC		3.42	2.73	0.78	0.75	0.39	0.37	0.30
YC		3.34	2.61	0.73	0.71	0.37	0.36	0.32
*SEM* ^2^		0.049	0.043	0.023	0.021	0.013	0.011	0.012
	C-	3.45	2.75 ^a^	0.78	0.76	0.39	0.37	0.31
	C+	3.32	2.60 ^b^	0.73	0.70	0.37	0.35	0.31
*SEM* ^2^		0.049	0.043	0.023	0.021	0.013	0.011	0.012
*p*-values								
Corn type × Coccidiosis		0.024	0.003	0.043	0.053	0.064	0.086	0.851
Corn type		0.276	0.055	0.137	0.239	0.243	0.351	0.148
Coccidiosis		0.077	0.022	0.162	0.073	0.259	0.112	0.781

^a,b^ Within each response variable, when the Corn type × Coccidiosis challenge interaction was significant (*p* ≤ 0.05), interaction least-squares means were separated using the LSMEANS/PDIFF option. When the interaction was not significant, main effects were interpreted from the corresponding least-squares means and GLM *p*-values. Means within the same effect and column with different superscripts differ significantly (*p* ≤ 0.05). YC = Yellow corn, OC = Orange corn; C+ = presence of coccidiosis challenge, C- = absence of coccidiosis challenge. ^1^ Values are least-squares means based on one sampled bird from each of 8 replicate pens per treatment. ^2^ SEM: Standard error of the mean for interactions and main effects.

**Table 3:** pH at 2 h, 24 h, and 5 mo, differences from corn diet treatments ^1^.

Factors		pH 2 h	pH 24 h	pH 5 mo
Corn Type	Coccidiosis Challenge			
		Interaction effects		
OC	C-	6.44	5.90	6.12
	C+	6.27	5.97	6.13
YC	C-	6.24	5.98	6.27
	C+	6.28	5.90	6.19
*SEM* ^2^		0.056	0.051	0.042
		Main effects		
OC		6.36	5.94	6.12 ^b^
YC		6.26	5.94	6.23 ^a^
*SEM* ^2^		0.039	0.036	0.030
	C-	6.34	5.94	6.19
	C+	6.28	5.94	6.16
*SEM* ^2^		0.040	0.036	0.030
*p*-values				
Corn type × Coccidiosis		0.106	0.156	0.314
Corn type		0.131	0.991	0.019
Coccidiosis		0.292	0.934	0.440

^a,b^ Within each response variable, when the Corn type × Coccidiosis challenge interaction was significant (*p* ≤ 0.05), interaction least-squares means were separated using the LSMEANS/PDIFF option. When the interaction was not significant, main effects were interpreted from the corresponding least-squares means and GLM *p*-values. Means within the same effect and column with different superscripts differ significantly (*p* ≤ 0.05). YC = Yellow corn, OC = Orange corn; C+ = presence of coccidiosis challenge, C- = absence of coccidiosis challenge. ^1^ Values are least-squares means based on one sampled bird from each of 8 replicate pens per treatment. ^2^ SEM: Standard error of the mean for interactions and main effects.

**Table 4:** Color at 24 h and 5 mo: differences from corn-diet treatments ^1^.

Factors		L*		a*		b*	
Corn Type	Coccidiosis Challenge	24 h	5 mo	24 h	5 mo	24 h	5 mo
		Interaction effects					
OC	C-	61.51	58.38 ^ab^	2.20	0.94	10.69 ^ab^	10.06 ^ab^
	C+	62.20	60.37 ^ab^	3.17	0.97	12.67 ^a^	11.41 ^a^
YC	C-	63.32	60.98 ^a^	2.22	−0.17	11.04 ^ab^	9.65 ^b^
	C+	60.89	57.54 ^b^	1.35	0.14	10.12 ^b^	8.91 ^b^
*SEM* ^2^		0.960	1.017	0.551	0.416	0.681	0.484
		Main effects					
OC		61.85	59.37	2.68	0.95 ^a^	11.68	10.73 ^a^
YC		62.10	59.26	1.78	−0.01 ^b^	10.58	9.28 ^b^
*SEM* ^2^		0.679	0.719	0.390	0.294	0.482	0.342
	C-	62.42	59.68	2.20	0.38	10.87	9.86
	C+	61.54	58.95	2.26	0.55	11.39	10.16
*SEM* ^2^		0.679	0.719	0.390	0.294	0.482	0.342
*p*-values							
Corn type × Coccidiosis		0.117	0.013	0.111	0.728	0.044	0.040
Corn type		0.795	0.913	0.115	0.029	0.121	0.006
Coccidiosis		0.371	0.483	0.928	0.686	0.448	0.542

^a,b^ Within each response variable, when the Corn type × Coccidiosis challenge interaction was significant (*p* ≤ 0.05), interaction least-squares means were separated using the LSMEANS/PDIFF option. When the interaction was not significant, main effects were interpreted from the corresponding least-squares means and GLM *p*-values. Means within the same effect and column with different superscripts differ significantly (*p* ≤ 0.05). YC = Yellow corn, OC = Orange corn; C+ = presence of coccidiosis challenge, C- = absence of coccidiosis challenge. ^1^ Values are least-squares means based on one sampled bird from each of 8 replicate pens per treatment. ^2^ SEM: Standard error of the mean for interactions and main effects.

**Table 5:** Distribution and mean severity scores of woody breasts among dietary corn and coccidiosis-challenge treatments ^1^.

Treatments	WB Scores				Means	n	p-Value
	0	1	2	3			
YC/C-	0	1	4	3	2.25 ^a^	8	0.013
OC/C-	1	1	4	2	1.88 ^ab^	8	
YC/C+	2	4	2	0	1.00 ^b^	8	
OC/C+	1	0	2	5	2.38 ^a^	8	
	*SEM* ^2^				0.328		

^a,b^ Least-squares means among corn type × coccidiosis challenge treatment combinations with different superscripts differ significantly based on LSMEANS/PDIFF (*p* ≤ 0.05). Woody breast severity scores were analyzed using a 2 × 2 factorial GLM including corn type, coccidiosis challenge, and their interaction. A Kruskal–Wallis test across the four treatment combinations was additionally conducted as a nonparametric sensitivity analysis and detected an overall treatment difference (χ^2^ = 9.93, df = 3, *p* = 0.0192). YC = Yellow corn, OC = Orange corn; C+ = presence of coccidiosis challenge, C- = absence of coccidiosis challenge. ^1^ Values are least-squares means from 8 replicate pens per treatment, with one bird sampled from each pen for woody breast assessment. ^2^ SEM: Standard error of the mean for the corn type × coccidiosis challenge interaction.

**Table 6:** Woody Breast Incidence percentage on each treatment from different corn diets ^1^.

Treatments		WB INCIDENCE %			
Corn Type	Coccidiosis Challenge	0	1	2	3
YC	C-	0	12.5	50	37.5
OC	C-	12.5	12.5	50	25
YC	C+	25	50	25	0
OC	C+	12.5	0	25	62.5

^1^ Percentages are based on 8 sampled birds per treatment, with one bird sampled from each replicate pen. The percentages presented in Table 6 are descriptive and were not subjected to a separate inferential analysis. WB = woody breast; YC = yellow corn; OC = orange corn; C+ = presence of coccidiosis challenge; C- = absence of coccidiosis challenge.

**Table 7:** Instrumental texture measurements of raw and cooked broiler breasts by Warner-Bratzler and BMORS methods ^1^.

Factors		Warner-Bratzler		BMORS					
Corn Type	Coccidiosis Challenge	Force (N)	Toughness (N·mm)	Shear Energy (N·mm)		Firmness (N)		Peak Count	
				Raw	Cooked	Raw	Cooked	Raw	Cooked
		Interaction effects							
OC	C-	33.78	428.99 ^a^	124.90 ^ab^	114.30	14.18 ^ab^	9.30	7.29	9.71
	C+	26.33	303.02 ^b^	150.21 ^ab^	115.13	17.60 ^ab^	9.79	6.62	7.71
YC	C-	30.09	315.27 ^b^	172.10 ^a^	153.63	19.75 ^a^	12.56	6.37	7.92
	C+	31.70	367.30 ^ab^	95.29 ^b^	99.57	10.73 ^b^	8.04	8.33	7.25
*SEM* ^2^		3.25	35.53	23.47	17.58	2.92	1.39	0.77	0.79
		Main effects							
OC		30.06	366.00	137.56	114.71	15.89	9.54	6.95	8.70
YC		30.89	341.29	133.69	126.60	15.24	10.30	7.35	7.58
*SEM* ^2^		2.50	29.07	4.84	3.52	0.64	0.33	0.27	0.61
	C-	31.94	372.13	148.50	133.97	16.96	10.93	6.83	8.81
	C+	29.01	335.16	122.75	107.35	14.17	8.91	7.47	7.47
*SEM* ^2^		2.50	29.07	4.84	3.52	0.64	0.33	0.27	0.61
*p*-values									
Corn type × Coccidiosis		0.177	0.019	0.039	0.131	0.043	0.085	0.103	0.407
Corn type		0.799	0.493	0.870	0.505	0.826	0.589	0.614	0.167
Coccidiosis		0.378	0.308	0.283	0.143	0.347	0.160	0.412	0.104

^a,b^ Within each response variable, when the Corn type × Coccidiosis challenge interaction was significant (*p* ≤ 0.05), interaction least-squares means were separated using the LSMEANS/PDIFF option. When the interaction was not significant, main effects were interpreted from the corresponding least-squares means and GLM *p*-values. Means within the same effect and column with different superscripts differ significantly (*p* ≤ 0.05). YC = Yellow corn, OC = Orange corn; C+ = presence of coccidiosis challenge, C- = absence of coccidiosis challenge. ^1^ Values are least-squares means based on one sampled bird from each of 8 replicate pens per treatment. ^2^ SEM: Standard error of the mean for interactions and main effects.

**Table 8:** Thaw, cook loss (%) and mg MDA/kg differences from corn diet treatments ^1^.

Factors		Thaw Loss	Cook Loss	mg MDA/kg
Corn Type	Coccidiosis Challenge			
		Interaction effects		
OC	C-	5.93	29.76	0.54
	C+	7.09	28.64	0.61
YC	C-	6.65	29.44	0.64
	C+	7.72	31.54	0.39
*SEM* ^2^		0.49	2.37	0.09
		Main effects		
OC		6.51	29.21	0.57
YC		7.18	30.49	0.52
*SEM* ^2^		0.34	1.67	0.06
	C-	6.29 ^b^	29.60	0.59
	C+	7.40 ^a^	30.10	0.50
*SEM* ^2^		0.34	1.67	0.06
*p*-values				
Corn type × Coccidiosis		0.934	0.504	0.079
Corn type		0.178	0.592	0.552
Coccidiosis		0.031	0.837	0.319

^a,b^ Within each response variable, when the Corn type × Coccidiosis challenge interaction was significant (*p* ≤ 0.05), interaction least-squares means were separated using the LSMEANS/PDIFF option. When the interaction was not significant, main effects were interpreted from the corresponding least-squares means and GLM *p*-values. Means within the same effect and column with different superscripts differ significantly (*p* ≤ 0.05). YC = Yellow corn, OC = Orange corn; C+ = presence of coccidiosis challenge, C- = absence of coccidiosis challenge. ^1^ Values are least-squares means based on one sampled bird from each of 8 replicate pens per treatment. ^2^ SEM: Standard error of the mean for interactions and main effects.

**Table 9:** Differences in meat composition among corn diet treatments ^1^.

Factors		Fat	Moisture	Protein	Collagen	Ash
Corn Type	Coccidiosis Challenge					
		Interaction effects				
OC	C-	2.60	75.72	20.46	0.57	1.69
	C+	2.20	76.95	19.54	0.57	1.71
YC	C-	2.94	75.84	19.77	0.62	1.72
	C+	2.21	75.91	20.76	0.63	1.71
*SEM* ^2^		0.22	0.39	0.45	0.05	0.01
		Main effects				
OC		2.40	76.34	20.00	0.57	1.70
YC		2.58	75.88	20.26	0.63	1.71
*SEM* ^2^		0.16	0.27	0.32	0.04	0.01
	C-	2.77 ^a^	75.78	20.12	0.60	1.70
	C+	2.20 ^b^	76.43	20.15	0.60	1.71
*SEM* ^2^		0.16	0.27	0.32	0.04	0.01
*p*-values						
Corn type × Coccidiosis		0.476	0.146	0.043	0.911	0.097
Corn type		0.419	0.249	0.569	0.219	0.229
Coccidiosis		0.018	0.110	0.947	0.960	0.396

^a,b^ Within each response variable, when the Corn type × Coccidiosis challenge interaction was significant (*p* ≤ 0.05), interaction least-squares means were separated using the LSMEANS/PDIFF option. When the interaction was not significant, main effects were interpreted from the corresponding least-squares means and GLM *p*-values. Means within the same effect and column with different superscripts differ significantly (*p* ≤ 0.05). YC = Yellow corn, OC = Orange corn; C+ = presence of coccidiosis challenge, C- = absence of coccidiosis challenge. ^1^ Values are least-squares means based on one sampled bird from each of 8 replicate pens per treatment. ^2^ SEM: Standard error of the mean for interactions and main effects.

## Data Availability

The original contributions presented in this study are included in the article/Supplementary Material. Further inquiries can be directed to the corresponding author.

## Supplementary Materials

The following supporting information can be downloaded at: https://www.mdpi.com/article/10.3390/foods15203607/s1, Supplementary Table S1. Ingredient composition (%) of experimental yellow corn (YC) and orange corn (OC) starter and grower diets; Supplementary Table S2. Analyzed nutrient composition of experimental yellow corn (YC) and orange corn (OC) starter and grower diets.
