# Supplementary material for: Effects of Dietary Corn Type and Coccidiosis Challenge on Broiler Meat Quality
Source: Foods. 2026 Oct 10;15(20):3607. doi: 10.3390/foods15203607 (PMC13704657; doi:10.3390/foods15203607)
Supplement: Supplementary file 1 [file foods-15-03607-s001.zip › foods-4572173-supplementary.pdf]

Supplementary Tables

Supplementary Table S1. Ingredient composition (%) of experimental yellow corn (YC) and orange corn (OC) starter and grower diets.

Ingredient (%)	YC Starter	OC Starter	YC Grower	OC Grower
Corn source[1]	55.99	55.70	66.45	66.34
Dehulled soybean meal	36.87	37.25	26.44	26.53
Vegetable fat	3.10	3.00	3.25	3.25
Dicalcium phosphate	1.51	1.51	1.17	1.17
Calcium carbonate	1.28	1.28	1.27	1.27
Salt (NaCl)	0.38	0.38	0.31	0.31
DL-Methionine	0.30	0.30	0.27	0.27
L-Lysine	0.25	0.25	0.35	0.35
L-Threonine	0.09	0.10	0.07	0.07
Choline chloride	0.08	0.09	0.09	0.09
Trace mineral premix	0.05	0.05	0.10	0.10
Vitamin premix	0.05	0.05	0.20	0.20
Phytase	0.02	0.02	0.02	0.02
Total	100.00	100.00	100.00	100.00

[1] Yellow corn was used in YC diets and orange corn was used in OC diets. Values are formulation inclusion percentages; totals reflect source-formulation rounding.

Supplementary Table S2. Analyzed nutrient composition of experimental yellow corn (YC) and orange corn (OC) starter and grower diets.

Analyzed component	YC Starter	OC Starter	YC Grower	OC Grower
Moisture (%)	11.94	13.06	12.62	13.12
Dry matter (%)	88.06	86.94	87.38	86.88
Crude protein (%)	21.80	22.20	17.90	18.80
Crude fat (%)	5.40	5.10	5.50	5.40
Acid detergent fiber (%)	3.20	3.50	2.60	2.40
Ash (%)	5.34	5.19	4.11	4.47
Metabolizable energy (Mcal/lb)	1.37	1.35	1.42	1.39
Sulfur, total (%)	0.27	0.27	0.27	0.22
Phosphorus, total (%)	0.70	0.68	0.61	0.57
Potassium, total (%)	1.01	1.05	0.88	0.81
Magnesium, total (%)	0.17	0.17	0.15	0.15
Calcium, total (%)	1.21	1.02	0.94	0.90
Sodium, total (%)	0.19	0.17	0.15	0.17
Iron, total (ppm)	185.0	177.0	171.0	305.0
Manganese, total (ppm)	63.4	48.8	89.4	74.8
Copper, total (ppm)	26.6	10.2	15.5	11.1

Zinc, total (ppm)	88.7	69.7	87.3	71.6

Values are reported on an as-received basis from finished-feed analyses. Metabolizable energy was calculated by the analytical laboratory. Mineral analyses were performed by ICAP using a wet-digestion procedure. YC = yellow corn; OC = orange corn.
